# Efficacy and safety of endovascular treatment in patients older than 90 with acute ischemic stroke: A retrospective cohort study

**DOI:** 10.3389/fneur.2022.1097423

**Published:** 2022-12-22

**Authors:** Itamar Friedman, Jonathan Naftali, Keshet Pardo, Michael Findler, Rani Barnea, Ran Brauner, Alin Perlow, Eitan Auriel, Guy Raphaeli

**Affiliations:** ^1^Sackler Faculty of Medicine, Tel Aviv University, Tel Aviv, Israel; ^2^Department of Neurology, Rabin Medical Center, Petach Tikva, Israel; ^3^Interventional Neuroradiology Unit, Rabin Medical Center, Petach Tikva, Israel

**Keywords:** stroke, endovascular treatment, elderly, nonagenarians, octogenarians

## Abstract

**Background:**

Endovascular treatment (EVT) for acute ischemic stroke (AIS) with large vessel occlusion (LVO) is the standard of care treatment today. Although elderly patients comprise the majority of stroke patients, octogenarians and non-agenarians are often poorly represented or even excluded in clinical trials. We looked at the safety and efficacy of EVT for AIS with LVO in patients over 90 (Non-agenarians), in comparison to patients aged 80–89 (Octogenarians) and to patients younger than 80 years (<80yrs).

**Methods:**

A retrospective analysis of patients who underwent EVT in a single stroke center during 2015–2019. Patients were divided into three subgroups based on their age: Non-agenarians, Octogenarians, and patients <80 yrs. The groups were compared based on baseline characteristics and stroke variables. In addition, we compared clinical and radiological outcomes including functional outcomes measured by the modified ranking scale (mRS) at day 90, symptomatic intracranial hemorrhage (sICH), and mortality.

**Results:**

Three hundred and forty seven patients were included, 20 (5.7%) of them were non-agenarians, 96 (27.7%) were octogenarians and 231 (66.6%) were <80 yrs. No statistically significant differences were found between groups regarding baseline characteristics, cardiovascular risk factors, stroke variables, or successful revascularization rates. Puncture to recanalization time intervals showed an age-related non-significant increase between the groups with a median time of 67.8, 51.6, and 40.2 min of the non-agenarian, octogenarian, and <80 yrs groups, respectively (*p*-value = 0.3). Favorable outcome (mRS 0–2) was 15% in non-agenarians vs. 13.54% in octogenarians (*p*-value = 1) and 40.2% in <80 yrs. sICH occurred among 5% of non-agenarians, compared to 4% among octogenarians (*p*-value = 1) and 2.6% in <80 yrs. The mortality rate at 3 months was significantly higher (55%) in non-agenarians compared to octogenarians (28%) (*p*-value = 0.03) and to <80 yrs (19.48%).

**Conclusion:**

EVT in nonagenarians demonstrated a high rate of successful revascularization, whilst also showing an increased rate of sICH when compared to octogenarians. Mortality rates showed an age-related correlation. Although further studies are needed to clarify the patient selection algorithm and identify sub-groups of elderly patients that could benefit from EVT, we showed that some patients do benefit from EVT therefore exclusion should not be based on age alone.

## Introduction

Acute Ischemic stroke (AIS) is one of the leading causes of morbidity and mortality worldwide (1–3). Treatment for AIS with large vessel occlusion (LVO) is primarily divided into intravenous thrombolysis (IV tPA), and endovascular thrombectomy (EVT) treatments (4), either by stent retrievers or catheter aspiration devices (5). Clinical randomized controlled trials (RCTs) demonstrated significantly better functional outcomes in patients treated with EVT, with or without prior administration of IV-tPA (4, 6–15).

Global average life expectancy continues to rise and the proportion of very elderly people (individuals aged 80 years or older) is expected to double in the next few decades (16, 17). Due to the inherent connection between cardiovascular and cerebrovascular diseases and age, which can be demonstrated by the fact that very elderly patients have the highest incidence and prevalence rates of AIS (1), the medical burden of these diseases is expected to reach new highs in the foreseeable future.

Despite that, RCTs often exclude elderly patients from their cohorts. Furthermore, their inclusion in clinical trials is often generalized and does not differentiate non-agenarians and octogenarians (18–21).

We aimed to explore the evidence basis of EVT in this challenging sub-group of patients, and further examine the effectiveness and safety of EVT for AIS with LVO in non-agenarians in comparison to octogenarians and to patients younger than 80 years.

Our main comparison was between the octogenarians and the non-agenarians, as the two groups had seldom been investigated, analyzed, or compared before. The third group (under 80 years of age) was used primarily to showcase the baseline results in young patients in our medical center, which shows similar outcomes as is common practice in comprehensive stroke centers worldwide.

## Methods

This is a retrospective analysis of a single center cohort, which includes all patients treated with EVT for AIS with LVO in Rabin medical center (RMC), Israel, between 2015 and 2019. We excluded patients who underwent spontaneous recanalization without intervention, and pre-stroke dependent functional status mRS ≥3. Patients were divided into three age sub-groups: Non [a]agenarians (aging ≥ 90 and <100), octogenarians (aging ≥80 and <90), and patients under 80 years of age.

We analyzed baseline and demographic characteristics, stroke characteristics including vascular territory and National institute of health stroke severity (NIHSS) score at admission, procedure endpoints such as good angiographic outcome (measured by mTICI score of 2c/3) and puncture to recanalization time interval, and patient outcomes such as Good clinical outcome measured by modified ranking scale (mRS) of ≤ 2 at 90 days, Symptomatic intracranial hemorrhage (sICH) Measured by, at least, four points increase in NIHSS score after 24 h compared to admission (22) and mortality. for all three groups. We compared our two main groups- nonagenarians and octogenarians, in regards to all variables, showing *p*-values correlating to that comparison alone.

The research was approved by the local Review Board.

### Statistical analyses

Data was analyzed by SAS program version 9.4. Continuous variables were described using average and standard deviation. Categorical variables were described using prevalence and percentages. The difference between the groups was tested using the ANOVA test for continuous variables and the Chi-squared test for categorical variables. Survival data was compared between the three groups using the Kaplan–Meier analysis.

## Results

A total number of 347 patients underwent EVT for LVO at our institution during the study period. Of them, 20 (5.7%) were non-agenarians, 96 (27.7%) were octogenarians and 231 (66.6%) were patients under 80 years of age (<80 yrs).

No statistically significant difference was found between the non-agenarian and octogenarian groups in regards to patient baseline characteristics (Table 1). When assessing the non-agenarians and octogenarians in comparison to <80 yrs, we found a slight difference of fewer current smokers, 5 and 4.17% in comparison to 28.57%, respectively. No significant association effect was found between mechanism of ischemic stroke [measured by TOAST (23) classification], vascular territory, onset to puncture time, NIHSS on admission, early ischemic changes on CT [ASPECT (24)], use of CT perfusion (CTP), angiographic collaterals status [ASTIN/SIR (25)] scores at admission, administration of IV-tPA or incidence of wake-up strokes (Table 2). The rates of good angiographic outcome, measured by modified treatment in cerebral infarction (mTICI) score of 2c/3, were similar in all three groups (70% in non-agenarians, 72% in Octogenarians and 65% in patients <80 yrs, *p* = 1). No significant difference was found when assessing the number of attempts for clot retrieval or median puncture to recanalization time interval (Table 3).

**Table 1:** Baseline characteristics.

	**Age group**			***P*-value**
	**<80 years (*n* = 231)**	**80–89 years (*n* = 96)**	**≥90 Years (*n* = 20)**	
Mean age in years (range)	65.2 ± 11.14 (22–79)	83.92 ± 2.63 (80–89)	92.6 ± 1.82 (90–95)	<0.01
**Gender**				
Male	136 (59%)	34 (35%)	4 (20%)	0.3
Female	95 (41%)	62 (65%)	16 (80%)	
**Cardiovascular risk factors**				
Hypertension	151 (65%)	74 (77%)	13 (65%)	0.27
Atrial fibrillation	*73 (32%)*	45 (47%)	10 (50%)	0.8
Diabetes	73 (32%)	33 (34%)	4 (20%)	0.3
Dyslipidemia	116 (50%)	51 (53%)	9 (45%)	0.6
Current smoking	66 (29%)	4 (4%)	1 (5%)	1
Ischemic heart disease	62 (27%)	28 (29%)	3 (15%)	0.3
Prior transient ischemic attack or stroke	50 (22%)	18 (19%)	5 (25%)	0.5
Prior carotid endarterectomy	3 (1%)	2 (2%)	2 (10%)	0.14

**Table 2:** Stroke characteristics.

		**Age group**			***P*-value**
		**<80 years** **(*n* = 231)**	**80–89 years** **(*n* = 96)**	**≥90 years (*n* = 20)**	
Mechanism of ischemic stroke (TOAST classification)	Large vessel atherosclerosis	58 (25%)	16 (17%)	5 (25%)	0.15
	Cardio-embolic	99 (43%)	59 (61%)	10 (50%)	
	Other	13 (6%)	2 (2%)	2 (10%)	
	Unknown	61 (26%)	19 (20%)	3 (15%)	
Vascular territory	Anterior	195 (84%)	89 (93%)	17 (85%)	0.37
	Posterior	36 (16%)	7 (7%)	3 (15%)	
Artery	Anterior cerebral artery	6 (3%)	2 (2%)	1 (5%)	0.43
	Middle cerebral artery	147 (65%)	67 (30%)	12 (5%)	0.43
	Posterior cerebral artery	7 (3%)	1 (1%)	1 (5%)	0.3
	Intra-cranial / T-Lesion	43 (19%)	20 (21%)	4 (20%)	0.87
	Extra-cranial / Tandem lesion	24 (10%)	8 (8%)	1 (5%)	
	Vertebral or basilar artery	29 (13%)	6 (6%)	2 (10%)	0.62
NIHSS score at admission, median (IQR)		15 (11–18)	15.5 (13–18.5)	17 (13.5–19)	0.43
ASPECT score at admission, median (IQR)		9 (7–10)	8.5 (7–9.5)	8.5 (7–10)	0.91
Performed CT perfusion		124 (54%)	59 (61%)	14 (70%)	0.42
ASITN/SIR score at admission, median (IQR)		3 (2–4)	4 (3–4)	3 (2–4)	0.23
Administration of IV-tPA		98 (42%)	31 (32%)	9 (45%)	0.31
Wake up stroke		56 (24%)	30 (31%)	7 (35%)	0.79

NIHSS, National Institutes of Health Stroke Scale; ASPECT, Alberta Stroke Program Early CT; ASITN/SIR, American Society of Interventional and Therapeutic Neuroradiology/Society of Interventional Radiology; tPA, Tissue plasminogen activator.

**Table 3:** Procedure endpoints.

	**Age group**			***p*-value**
	**<80 years (*n* = 231)**	**80–89 years (*n* = 96)**	**≥90 years (*n* = 20)**	
Good angiographic outcome (mTICI score of 2c/3)	151 (65%)	69 (72%)	14 (70%)	1
Number of attempts for clot retrieval, median (IQR)	1 (1–3)	2 (1–3)	2 (1–3)	0.69
Onset to puncture time interval (minutes), median (IQR)	295.2 (220.2–394.8)	303 (229.8–445.2)	300 (270–405)	0.73
Puncture to recanalization time (minutes), median (IQR)	40.2 (25.2–70.2)	51.6 (30–97.2)	67.8 (41.4–103.2)	0.33

mTICI, modified treatment in cerebral ischemia.

**Figure 1:**
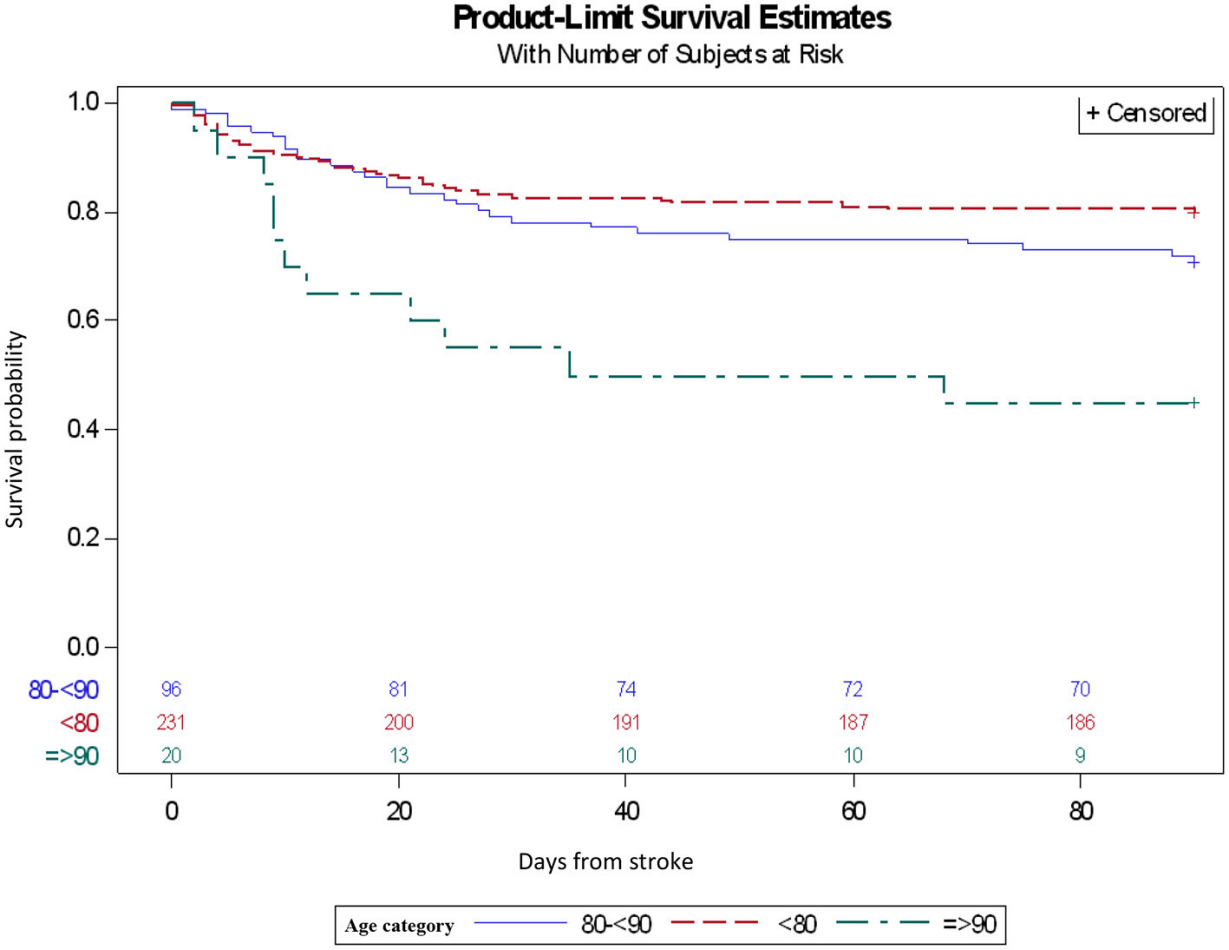
Kaplan-Meier plot of mortality rates at 90 days by age group.

We did not find a difference in good clinical outcome (measured as mRS score of ≤ 2 at 90 days) between non-agenarians and octogenarians (15 vs. 13.54%, *p*-value = 1), however we did find a notable difference compared to the <80 yrs group, with an average of 40.26% (Table 4). Early neurological improvement (measured by either a drop of at least 10 points in NIHSS score, or NIHSS score of 0–1, 24 h after procedure) rates were 20% in non-agenarians vs. 19.79% in octogenarians (. = 1), and 26.41% in patients <80yrs. Symptomatic ICH was observed in 5% of non-agenarians vs. 4.17% of octogenarians (*p* = 1), almost twice greater when compared to patients <80 years of age (2.6%). Mortality rates at 90 days were significantly higher among nonagenarians (55%), a 2-fold increase compared to octogenarians (28.13%, *p* = 0.03) and almost 3-fold compared to <80 yrs (19.48%). This trend can be clearly seen in the Kaplan-Meier plot (Figure 1). The Non-agenarian survival rate is significantly poorer throughout the whole follow-up period, while octogenarians and <80 yrs group show similar survival patterns.

**Table 4:** Patient outcomes.

	**Age group**			***p*-value**
	**<80 years (*n* = 231)**	**80–89 years (*n* = 96)**	**≥90 years (*n* = 20)**	
Good Clinical outcome (mRS score of ≤ 2 at 90 days)	93 (40.26%)	13 (13.54%)	3 (15%)	1
Early neurological improvement^*^	61 (26.41%)	19 (19.79%)	4 (20%)	1
Symptomatic Intracerebral hemorrhage	6 (2.6%)	4 (4.17%)	1 (5%)	1
Mortality rate at 90 days	45 (19.48%)	27 (28.13%)	11 (55%)	0.03

mRS, modified Rankin Scale; NIHSS, National Institutes of Health Stroke Scale.

* Measured by either a decrease of at least 10 points in NIHSS score, or NIHSS score of 0/1, 24 h after recanalization.

## Discussion

Our aim was to assess efficacy and safety of EVT in non-agenarians compared to octogenarians. While most of our patients were younger than 80 years, 5.7% of our patients were non-agenarians and 27.7% octogenarians. To summarize outcomes, successful revascularization was achieved in 70% (similar to octogenarians); favorable outcome (mRS 0–2) was 15% (13.54% octogenarians), which is significantly lower in comparison to <80 years (40.2%). Mortality rate at 3 months was significantly higher (55%) in non-agenarians compared to octogenarians (28%). In terms of safety, sICH occurred amongst 5% of non-agenarians, compared to 4% in octogenarians.

Our findings are similar to those reported previously, including the important systemic review and meta-analysis published by Bai et al. (26) in 2021, where they showed good clinical outcome rates of 21.6%, successful revascularization rates of 80.82%, sICH rates of 3.52% and a 3 month mortality rate of 44.38%. Their conclusion was that MT in non-agenarians shows a high rate of successful revascularization, while simultaneously resulting in a high rate of low functional independence proportion and increased mortality, thus requiring careful selection of patients in order to achieve better results in the future (26).

To further establish our results, we compared them to other cohorts. In a multicenter cohort, Khan et al. (27) compared 18 nonagenarian patients to 175 patients <90 years of age, and showed very similar results regarding good clinical outcome (with 11.1 vs. 48%, respectively). Similarly, Alawieh et al. (28) reviewed over 560 patient files in a single center cohort and compared the results of 108 patients ≥80 years of age, 15 of them non-agenarians, with younger counterparts and reported similar results with 20.5 vs. 44.4% respectively.

We encountered similar rates of successful recanalization and onset-to-puncture time intervals in all three study groups, indicating a lack of major age-related differences. However, a major age-related difference was noticed regarding puncture-to-recanalization time interval, with a median time of 67.8, 51.6, and 40.2 min in the non-agenarian, octogenarian and <80 yrs groups, respectively. This difference might be explained by increased vessel tortuosity, challenging anatomy (including aortic arch) and vessel wall low compliance, which can hamper arterial access and intracranial navigability and traceability as shown in previous studies (19, 27, 29).

Although only in low numbers, we experienced similar sICH rates in both elderly groups, which were almost 2-fold compared to the <80 yrs group (5 vs. 4.17 vs. 2.6%, respectively). These results are similar to those reported in previous studies (28, 30, 31) and might be, at least, partially explained by the high incidence of reperfusion injury post recanalization in elderly patients.

Concerning mortality, we observed a statistically significant increase in the non-agenarian group - 55 vs. 28.13% in the octogenarian group (*p* = 0.03) and 19.48% in the <80yrs group. Sussman et al. (30), compared the results of 29 non-agenarians vs. 79 octogenarians who went through EVT in a 2019 single center cohort and found similar 90-day mortality rates with 63 and 40.9% (*p* = 0.07) in non-agenarians and octogenarians, respectively. When comparing elderly patients over 80 years of age with younger counterparts, Alawieh et al. (28) published similar findings with mortality rates of 34.3 vs. 20%, *P* < 0.001 in favor of younger patients. Our findings correlate well with these and other previous publications and help establish a clear trend of higher age-related mortality rate (26). Although sICH and mortality rates both share this age-related trend, we cannot conclude that there is a direct cause-effect relationship between the two, due to the small size of our cohort which inhibits our ability to achieve statistical significance.

When evaluating patient's eligibility for MT for LVO, our common practice, besides assessing the time window, is to review the patient's ASPECT score, CTP data and functional baseline rather than age. Therefore, it was unsurprising that no differences were found between the non-agenarian and octogenarian groups regarding these parameters (e.g., ASPECT and CTP prevalence). However, the results definitely emphasize the need for improving the patient selection process in the non-agenarian age group.

In light of the fact that we were not able to show non-inferiority in the non-agenarian group regarding good clinical outcome in 90 days, sICH incidence or mortality at 90 days, the inclination might be to claim that EVT for AIS in non-agenarian is less safe and that the risk/benefit ratio in these procedures is not favorable for this important sub-population. However, a general statement such as this must take into account all aspects of the equation, with an emphasis on the natural history of AIS, especially in elderly patients, which is well-documented and shows catastrophic results both in terms of morbidity, disability and mortality (2, 11, 32).

### Our study has some limitations

Due to the study design, being a retrospective cohort with a small sample group, there is some difficulty drawing conclusions on the general population. Moreover, information bias- due to retrospective data collection and the possibility of missing data from patients' files. Lastly, Selection bias- this cohort represents only patients treated by EVT and does not include all other patients who presented with AIS and LVO during the study period.

## Conclusion

EVT in non-agenarians demonstrated a high rate of successful revascularization. Non-agenarians might be at higher risk of sICH than octogenarians, despite similar stroke and treatment-related variables. Mortality rates at 90 days showed an age-related correlation which was statistically significant, and while there was a trend toward poor functional outcomes in non-agenarians, the difference was not statistically significant in this relatively small retrospective study.

In our opinion, MT is a valuable yet imperfect tool in the octogenarian and especially in the non-agenarian age groups, and should be considered on a case-by-case basis. We believe that treatment should not be withheld due to age alone as a sole criterion. However, future prospective studies are needed to find an adjusted patient selection algorithm for this population.

## Data availability statement

The datasets presented in this article are not readily available because of ethical and privacy restrictions. Requests to access the datasets should be directed to GR, graphaeli@gmail.com.

## Ethics statement

The studies involving human participants were reviewed and approved by Rabin Medical Center Helsinki Committee. Written informed consent for participation was not required for this study in accordance with the national legislation and the institutional requirements.

## Author contributions

IF: study design, data collection, data analysis, writing of manuscript, and submission of manuscript. JN, MF, and EA: study design and review of manuscript. KP and RBa: study design. RBr: proceduralist, study design, and review of manuscript. AP: proceduralist and study design. GR: proceduralist, study design, data collection, data analysis, review, and approval of manuscript. All authors contributed to the article and approved the submitted version.
